# Predicting Multiple Outcomes Associated with Frailty based on Imbalanced Multi-label Classification

**DOI:** 10.1007/s41666-024-00173-6

**Published:** 2024-10-02

**Authors:** Adane Nega Tarekegn, Krzysztof Michalak, Giuseppe Costa, Fulvio Ricceri, Mario Giacobini

**Affiliations:** 1 Department of Information Science and Media Studies, University of Bergen, Bergen, Norway; 2 Department of Information Technologies, Wroclaw University of Economics and Business, Wroclaw, Poland; 3 Department of Clinical and Biological Sciences, University of Turin, Turin, Italy; 4 Faculty of Computing, Bahir Dar Institute of Technology, Bahir Dar University, Bahir Dar, Ethiopia; 5 Data Analysis and Modeling Unit, Department of Veterinary Sciences, University of Turin, Turin, Italy

**Keywords:** Frailty prediction, Hybrid resampling, Imbalanced data, Multi-label classification, Resampling algorithm

## Abstract

Frailty syndrome is prevalent among the elderly, often linked to chronic diseases and resulting in various adverse health outcomes. Existing research has predominantly focused on predicting individual frailty-related outcomes. However, this paper takes a novel approach by framing frailty as a multi-label learning problem, aiming to predict multiple adverse outcomes simultaneously. In the context of multi-label classification, dealing with imbalanced label distribution poses inherent challenges to multi-label prediction. To address this issue, our study proposes a hybrid resampling approach tailored for handling imbalance problems in the multi-label scenario. The proposed resampling technique and prediction tasks were applied to a high-dimensional real-life medical dataset comprising individuals aged 65 years and above. Several multi-label algorithms were employed in the experiment, and their performance was evaluated using multi-label metrics. The results obtained through our proposed approach revealed that the best-performing prediction model achieved an average precision score of 83%. These findings underscore the effectiveness of our method in predicting multiple frailty outcomes from a complex and imbalanced multi-label dataset.

## Introduction

Frailty is a common clinical condition used to describe older people who are more vulnerable to stressors and therefore have a higher risk of negative health outcomes. It has been shown that frailty has become a major challenge in the modern society due to the aging population. Several definitions have been proposed in the literature to conceptualize and operationalize frailty [1, 2]. However, a universally accepted definition of frailty is still lacking, making it difficult to effectively target community services to older adults. Despite its challenges, frailty is not an irreversible process and can be reversed or delayed from its progression. Therefore, it is argued that it should be detected early. The frailty detection framework presented in this article highlights two major issues: (1) addressing the problem of highly imbalanced data in multi-label classification, and (2) predicting multiple adverse outcomes associated with frailty from a balanced multi-label dataset. This section presents first frailty and its prevalence in elderly people. Then, frailty is framed as a multi-label problem, and finally, key points that summarize the contents of this paper are stated.

### Prevalence of Frailty

Frailty is a dynamic and multidimensional clinical condition related to ageing, characterized by a decreased ability to maintain homeostasis and perform the normal activities of daily life [1, 2]. It is commonly recognized that frailty aggravates the risk of negative health outcomes (e.g., hospitalization, functional impairment, loss of autonomy, and death) and that it escalates health and social challenges [3]. Several studies [1–5] indicate that frailty has become one of the most serious health issues putting a heavy burden on elderly care systems. Its prevalence is expected to rise rapidly with the increasing number of older adults in almost all countries. In recent years, frailty has received increasing scientific attention as it has a significant influence on the quality and independence of life of older adults and available medical healthcare resources. There are several tools that have been used for the detection of frailty. The Fried Phenotypic Model [4] is one of the most widely used tools for assessing physical frailty. It is based on the quantification of five measurable components: self-reported exhaustion, slow walking speed, low grip strength, unintended weight loss, and low physical activity. According to this model, frailty is present if a person has at least three of the above pre-defined components. Following the concept of the Fried model, several studies were conducted to estimate the prevalence of frailty in older adults [5].

However, it should be noted that there is still considerable uncertainty around the concept of frailty [5, 6] and that a phenotypic evaluation of subjects is impossible when considering a large population. There have been several reasons why it is so difficult to define and conceptualize frailty, including its complex aetiology [7], the often-independent work of researchers in diverse areas of frailty, such as biological basis, social basis, environment, and technology [8, 9], and the inherent difficulty in distinguishing frailty from ageing and disability [10]. There also exists a considerable degree of heterogeneity among the different studies of frailty models in terms of sample type and size, population characteristics and settings, baseline frailty status, and outcomes. In general, the current challenges of frailty research include the lack of a standard definition of frailty, which leads to the lack of a standard screening and diagnostic tool, further understanding of interventions to reverse frailty, the best time for intervention, common understanding model to face the challenges and early estimation of multiple adverse outcomes in a frail patient [11, 12].

### Framing Frailty as Multi-label Problem

Until now, state-of-the-art statistical or analytical considerations have been targeted at the intervention of a single outcome or risk factor associated with frailty. For example, Fried’s frailty phenotype was specified as a serious risk factor for six-month mortality but was not linked with delirium and in-hospital falls [13]; similarly, a frailty risk model proposed in [14] was designed to predict in-hospital mortality for post cardiovascular surgery patients admitted to the intensive care unit. Bertini et al. [15] developed a model to predict all-cause mortality within a year. Other previous researches on frailty [16, 17] have also been focused on single-outcome prediction, where separate models were developed for predicting mortality, hospitalization, fracture, and disability.

Clinically, however, it is more important to make interventions on more than one simultaneous outcome with common heterogeneous risk factors associated with frailty. This is due to the fact that the co-existence of multiple chronic conditions or comorbidity is common in older people [18, 19], which contributes to multiple adverse outcomes. Therefore, this study aims to build a predictive model that considers the correlation among multiple outcomes to provide a list of relevant outputs for a previously unseen patient. In this case, we frame frailty as a multi-label problem and developed a multi-label classification (MLC) model to predict the six outcomes of frailty simultaneously: mortality, urgent hospitalization, disability, fracture, medical emergency admission at the emergency department, and preventable hospitalization.

MLC is focused on training prediction functions that can associate an instance with multiple labels that are not necessarily mutually exclusive [20]. These days, MLC has gained considerable attention in the machine learning community. It appears in many application domains, and it is natural for many real-world problems, such as clinical diagnosis, disease prediction, activity recognition, object detection, image classification, etc. The existing methods for the MLC task are algorithm adaptation and problem transformation methods. The former transforms the MLC task into one or more single label classification tasks [21], regression problems, or label ranking [22] tasks, while the latter could extend specific learning algorithms to handle multi-label datasets directly [23].

#### Imbalanced Problem in MLC

In any machine learning task, the problem of imbalanced classification is among the factors that pose significant challenges in the training process of a learning model. A recent and comprehensive review of methods for addressing imbalanced problems in multi-label classification is presented in [24]. An imbalanced problem is an inherent and well-known characteristic of most multi-label datasets. Three types of imbalance problems can be present in an MLC [24]. These are (1) imbalances between labels, where there is unequal frequency or distribution of labels in a multi-label dataset (MLD) [25]. As each sample of an MLD is usually correlated with multiple labels, some of them can be majority labels while others are minority ones. (2) imbalance within labels that occurs when at least one label contains a smaller number of positive samples, and a greater number of negative samples [26], and (3) imbalance among the label sets, where more frequent sets of labels and rare sets of labels exist in an MLD [27].

Among these three types of imbalances, the presence of imbalanced classification between labels in an MLC is the most challenging one in which one label may contain a much larger number of 1’s than the other label. Such imbalanced label distributions are the intrinsic characteristics of most multi-label datasets. More specifically, the majority and minority labels may occur jointly in the same instances that affect the prediction performance of multi-label learning methods. In this study, a hybrid of resampling methods is proposed to reduce the imbalanced label distributions while also reducing the imbalance between classes in each label.

#### Hybrid Resampling Approaches for MLC

In single-label learning (or standard classification), it is a common practice to use the synthetic minority oversampling technique (SMOTE) [28], Tomek links (T-link) [29] or their hybrid version. However, the imbalanced problem in MLC is much more complicated than in single-label classification due to the presence of imbalance within labels, among label sets, and between labels altogether. In this study, we extend systematically the use of single-label resampling approaches (SMOTE and T-link) for the multi-label scenario that reduces the problem of imbalance among labels and within the labels. In the multi-label scenario, SMOTE produces a set of samples, where each minority label occurs. Each minority instance will be the seed (i.e., used as a reference point) for a new synthetic sample. The set of features and label sets appearing in the reference instances will also be added for the new instances. The hybrid version of SMOTE and T-link is used in our multi-label problem to avoid the imbalance between labels as well as to reduce the imbalance between classes in each label. The set of majority and minority labels is identified based on the imbalance level measures [30].

### Highlights of this Study

Existing methods, such as SMOTE and Tomek, have been developed to handle the imbalance problem in the traditional single-label classification. Unfortunately, these single-label approaches fail to work in the multi-label learning problem due to the presence of more than one label associated with an instance of the data. This paper presents an extension of the single-label approach to handling imbalanced multi-label classification while predicting multiple outcomes associated with frailty. To the best of our knowledge, this work is the first to apply MLC strategies to the frailty problem and to propose a novel hybrid resampling approach to address the intrinsic problem of imbalanced data in the multi-label learning problem. This innovative approach demonstrates the practical utility and relevance in a real-world healthcare setting, highlighting its originality and importance. The key points summarizing the contents of this article include:

- This article addresses the prediction of multiple adverse outcomes associated with frailty based on a highly imbalanced multi-label clinical dataset.
- The core challenge in our dataset is the joint occurrence of more frequent and less frequent labels in the same sample, which cannot directly be solved by the existing single-label resampling approaches, such as oversampling, and undersampling methods.
- The proposed hybrid resampling approach presented in this article aims to solve the problem of imbalance in multi-label classification, which is strongly motivated by the practical problem of predicting several outcomes associated with frailty from an imbalanced multi-label dataset.
- The proposed method significantly improves the classification performance of multi-label algorithms in predicting simultaneous outcomes.

## Background and Preliminaries

In this section, we provide a brief introduction to the concept of MLC, imbalanced MLC, imbalance quantification methods, evaluation metrics, and single-label resampling approaches.

### Multi-Label Classification

MLC problem is a generalization of a single-label (binary or multi-class) classification problem where an instance is associated with more than one label simultaneously. In this study, the frailty risk prediction problem is formulated as a multi-label classification problem. Given a set of $m$ medical records $M=\left\{{r}_{1},{r}_{2}, . . .,{r}_{m}\right\}$ and a finite set of $q$ outcomes$L=\left\{{\lambda }_{1},{\lambda }_{2}, . . .,{\lambda }_{q}\right\}$, each record in $M$ is associated with one or more outcomes in$L$. In this context, the ‘outcomes’ represent the labels. The set of multi-label training examples of the frailty classification problem can be represented by$S=\left\{\left({r}_{i},{Y}_{i}\right) , i=1,. . .,m\right\}$, where ${r}_{i}$ is the feature vector and ${Y}_{i}\subseteq L$ denotes the set of labels for the ${i}^{th}$ record. The objective is to build a classification model to predict a set of labels ${\widehat{Y}}_{i}$ for every new record${r}_{i}{\prime}$. In this study, for any patient, multiple outcomes were identified in the data, and each outcome is considered as a label.

There are several multi-label classification strategies to train an MLD [31]. The most widely used and straightforward approach is binary relevance (BR). It considers each label as an independent problem and trains one binary classifier per label. BR is the baseline MLC algorithm that does not consider the relationships that may exist between labels. To overcome this limitation, several ensemble approaches, such as classifier chains (CC) and label powersets (LP), have been proposed. CC extends BR by taking some label correlation into account. It works by feeding the predictions of earlier classifiers as features to the latter classifier. However, the CC algorithms suffer from the issue of label ordering, as classifiers with different chain positions receive different levels of information. LP-based classifiers use subsets of label-sets as class identifiers where each unique set of labels for an MLD is considered as a single label. On datasets with a large number of label combinations, LP has the drawback of ending up with a large number of represented classes and few samples to train on. Random k-label-set (RAkEL) [32] is an improvement to avoid the problem of the LP method within a large number of unique label sets. It builds classifiers that are the ensemble of LP, and every LP model is trained on a different smaller subset of labels. The class labels are then determined by a voting procedure based on a threshold. The RAkEL approach takes label correlation into account and has lower complexity than the LP method. All three approaches (BR, CC, and LP) are grouped under problem transformation methods, where the MLC problem is transformed into a binary or multi-class problem.

Ranking by pairwise comparison (RPC) [33] creates a pairwise transformation of the multi-label dataset into $\frac{\left|L\right|\left(\left|L\right|-1\right)}{2}$ binary problems, one for each pair of labels (${\lambda }_{i},{\lambda }_{j}$), $1\le i<j\le L$. On each dataset, a model is trained based on examples annotated by exactly one of the labels, but not both. Calibrated label ranking (CLR) [22] extends RPC by initiating one supplementary virtual label, which indicates the boundary (separation point) between relevant and irrelevant labels. When classifying a new sample, each binary classifier is invoked to vote and predict one of the two labels. Finally, classifiers are evaluated, and the labels are ranked according to their sum of votes. This way, it manages to solve both the MLC and MLR (multi-label ranking) tasks. MLkNN (Multi-label K nearest neighbours) is an adaptation method of the K nearest neighbours (KNN) algorithm to a multi-label problem [23]. MLkNN uses the same basic principle as KNN, except that MLkNN uses a Bayesian approach of prior probability and posterior probability to specify the relevant label sets. These MLC models are well-suited for modeling multiple labels simultaneously, unlike traditional classification, which are designed to predict a single output [34, 35].

### Imbalance Quantification Methods

The imbalance quantification method designed for the single-label (binary /multi-class) classification assumes the ratio of minority to majority class as an imbalance measure, which is not suitable for multi-label classification. Learning from an imbalanced MLD is a more complex problem in MLC due to the large label space when considering all possible label combinations. Various measures can be used to quantify the level of imbalance in MLC. In this study, we use the following measures to identify minority and majority labels [27].

#### IRLbl (Imbalance ratio per label)

Given a set of labels $L$ and ${Y}_{i}$ be the label set of the ${i}^{th}$ sample in M (M is an MLD), IRLbl is calculated for a label λ as the ratio between the most frequent label and the label λ. The most occurring label has an IRLbl of 1 and a higher value for the rest.

1 $$IRLbl\left(\lambda \right)=\frac{\underset{{\lambda }^{'}\in\; L}{\mathit{max}}\,(\sum_{i=1}^{m}h({\lambda }^{{'}},{Y}_{i})) }{\sum_{i=1}^{m}h(\lambda ,{Y}_{i})) } , h\left(\lambda ,{Y}_{i}\right)=\left\{\begin{array}{c}1 \lambda \in {Y}_{i}\\ 0 \lambda \notin {Y}_{i}\end{array}\right.$$

#### MeanIR (Mean imbalance ratio)

MeanIR can be computed as the mean imbalance ratio of all labels in an MLD.

2 $$MeanIR=\frac{1}{q}\sum\nolimits_{\uplambda \in \text{L}}IRLbl\left(\lambda \right)$$

#### MaxIR (Maximum imbalance ratio)

It is the proportion of the most common label to the rarest one.

3 $$MaxIR=\underset{\lambda \in\; L}{\mathit{max}}\left(IRLbl\left(\lambda \right)\right)$$

#### CVIR (Coefficient of variation of IRLbl)

CVIR measures the variation of IRLbl, i.e., the similarity of the level of imbalance between all labels. It indicates if labels experience a similar level of imbalance or, on the contrary, there are large differences among them. The higher the CVIR value, the higher would be this difference:

4 $$CVIR=\frac{IRLbl\sigma }{MeanIR} , IRLbl\sigma =\sqrt{\sum\nolimits_{\lambda \in L}\frac{{\left(IRLbl\left(\lambda \right)-MeanIR\right)}^{2}}{q-1}}$$

As it is declared in [27], the joint use of MeanIR and CVIR measures represents whether an MLD is imbalanced or not, while IRLbl is important to evaluate the imbalance level of each label. An MLD with a MeanIR value higher than 1.5 and a CVIR value greater than 0.2 should be considered imbalanced.

### Evaluation Metrics for MLC

The evaluation of models in MLC differs from the traditional single-label classification. It requires a special approach in order to consider performance over all labels. In this study, the average precision, Hamming loss, ranking loss, F-score micro averaged, and area under the ROC curve (AUROC) macro average were used to evaluate the performance of different MLC models. To formally define each evaluation measure, consider the instances of an MLD $\left({x}_{i},{Y}_{i}\right), i=1, . . . , m,$ where ${Y}_{i}\subseteq L$ is the set of true labels and$L=\left\{{\lambda }_{1},{\lambda }_{2}, . . .,{\lambda }_{q}\right\}$, is the label space. For a given sample${x}_{i}$, the set of predicted labels by the MLC model is denoted by${Z}_{i}$, and the rank that is predicted by a label ranking method for a label λ is represented by *ri*(λ).

**Average precision (**AP**)** computes the proportion of labels ranked ahead of a certain label λ∊${\text{Y}}_{\text{I}}$ which actually are in ${\text{Y}}_{\text{i}}$. AP allows knowing the percentage of correct positive predictions.

5 $$\text{AP}={\frac{1}{\text{m}}}_{ }{\sum\nolimits }_{\text{i}=1}^{\text{m}}\frac{1}{\left| {\text{Y}}_{\text{I}} \right| }\sum\nolimits_{\lambda \in {Y}_{i}}\frac{\left|\left\{{\lambda }^{\prime}\in {Y}_{i}:ri\left({\lambda }^{\prime}\right)\le ri\left(\lambda \right)\right\}\right|}{ri\left(\lambda \right)}$$

**Hamming loss (HL)** is the commonly used evaluation metric in MLC, calculated as the difference between the true and predicted labels divided by the sum of all of the labels in the MLD [36]. The score lies between 0 and 1, where 0 is the best.

6 $$\text{HL }=\frac{1}{m}{\sum }_{i=1}^{m}\frac{\left| {Y}_{i} \Delta {Z}_{i}\right|}{\left|L\right|}$$

The symbol Δ represents the symmetric difference between the two sets. HL measures how many times, on average, an observation label set is misclassified. In this paper, HL is used to measure the capability of the algorithm to identify the presence of frailty in terms of adverse health outcomes.

**Ranking loss (RL)** measures how many times a relevant label (a member of the true label set) appears ranked lower than a non-relevant label. The score lies between 0 and 1, where 0 is the best:

7 $$\text{RL }={\frac{1}{\text{m}}}_{ }{\sum }_{\text{i}=1}^{\text{m}}\frac{1}{\left| {\text{Y}}_{\text{I}} \right| \left| {\overline{\text{Y}} }_{\text{i}}\right|}|\left\{\left({\lambda }_{a},{\lambda }_{b}\right) :ri\left({\lambda }_{a}\right)>ri\left({\lambda }_{b}\right), \left({\lambda }_{a},{\lambda }_{b}\right)\in {\text{Y}}_{\text{i}}x{\overline{\text{Y}} }_{\text{i}}\right\}|,$$

where ${\overline{Y} }_{I}$ is the complementary set of ${Y}_{I}$ with respect to L.

In the context of this study, RL is used to measure how well the algorithm ranks labels, which allows an understanding of the type of patient outcomes that have a strong expression, indicating where to act on time.

**Label-based metric** is computed for the labels by using micro and macro averaging [37]. Macro averaging can be calculated on each label independently followed by averaging over all the obtained values, while micro-averaging can be calculated over all the samples and class labels. Macro and micro-averaged measures for the area under the ROC (AUROC) and F1 score can be calculated as follows:

8 $$\begin{array}{c}AUROC_{macro}=\frac1q\sum_{\lambda\in L}\frac{\left|\left\{x{'},x{''}:rank(x',\;L_\lambda)\;\geq\;rank(x{''},\;L_\lambda),\;(x{'},\;x{''})\in X_\lambda\times\overline{X_\lambda}\right\}\right|}{\left|X_\lambda\right|.\left|\overline{X_\lambda}\right|}\\,X_\lambda=\left\{x_i\vert L_\lambda\in Y_i\right\},\overline{X_\lambda}=\left\{x_i\vert L_\lambda\not\in Y_i\right\}\\\end{array}$$

The function $rank({x}_{i},\lambda )$ is defined such that for a given instance ${x}_{i}$ and label $\lambda \in L$, where the position of $\lambda$ is known, it returns a confidence level of $\lambda$ in the prediction ${Z}_{i}$ made by the classifier.

9 $${{F}_{1}}_{micro}=\frac{\sum_{i=1}^{m}\sum_{\lambda =1}^{q}{Z}_{i}\times {Y}_{i} }{\sum_{i=1}^{\text{m}}\sum_{\lambda =1}^{\text{q}}{\widehat{Y}}_{i} + \sum_{i=1}^{\text{m}}\sum_{\lambda =1}^{\text{q}}{Y}_{i}}$$

where ${Z}_{i}$ and ${\text{Y}}_{\text{i}}$ are the predicted and actual values, respectively, for label $\lambda$ and instance $i$.

### Single-label Resampling Approaches

This section presents the essential background information on two resampling approaches: Tomek Links and Synthetic Minority Oversampling Technique. These methods have been widely used in addressing the challenge of imbalance in the single-label classification problem.

#### Tomek Links

Tomek links [29], T-link for short, is an enhancement of the nearest neighbour rule [38], which heuristically removes only the noisy or boundary instances of the two classes. The basic idea of the T-link algorithm is as follows:

1. Let i be an instance of class A and j be an instance of class B.
2. Let d(i, j) be the distance between i and j.
3. (i,j) is a T-link, if for any instance m ≠ i,j, d(i,j) < d(i,m) or d(i,j) < d(j,m). If any two examples are T-links, then one of the instances is noise, or both instances are located at the border of the class.
4. Remove noise or border points.
5. Repeat steps 1 to 3 until all possible pairs of classes are processed.

For a dataset with two target class values, a T-link is a pair of samples that are (1) nearest neighbours of one another, and (2) have different target class values [29]. Instances that belong to T-link pairs are likely to be either noise points or points that lie close to the optimal decision boundary. Eliminating those points can result in more well-defined class groups in the training data, which can lead to better classification [39]. T-link could be used as an under-sampling technique or as a post-process cleaning step [40]. If it is used as an under-sampling technique, only the samples from the majority class are removed. If it is used as a post-process cleaning step, samples from both the majority and minority classes are removed.

#### Synthetic Minority Oversampling

The Synthetic Minority Oversampling (SMOTE) [28] technique is an oversampling method where a minority class is oversampled to generate new instances using an interpolation technique. The basic idea is to create new samples that are located anywhere on the line that joins together each of the minority class samples and all (or some) of its k nearest neighbours (KNN). KNN uses the Euclidean distance function as the distance metric. The synthetic samples in SMOTE are generated using the following steps:

1. Choose the feature vector of the current sample (minority class sample).

2. Calculate its *k* nearest neighbours and randomly select the feature vector of one of these nearest neighbours.

3. The new instances are generated by interpolation technique (e.g., the difference between the selected sample’s feature vector and its selected nearest neighbour).

4. Multiply the result obtained in step 3 with a random value between 0 and 1 and add this vector to the feature vector of the current sample. This causes the selection of a random point along the line segment between two specific feature vectors.

5. The new vector will be the synthetic sample. Repeat these steps until the required number of instances to be generated is reached.

## Proposed Methodology

**Fig. 1:**
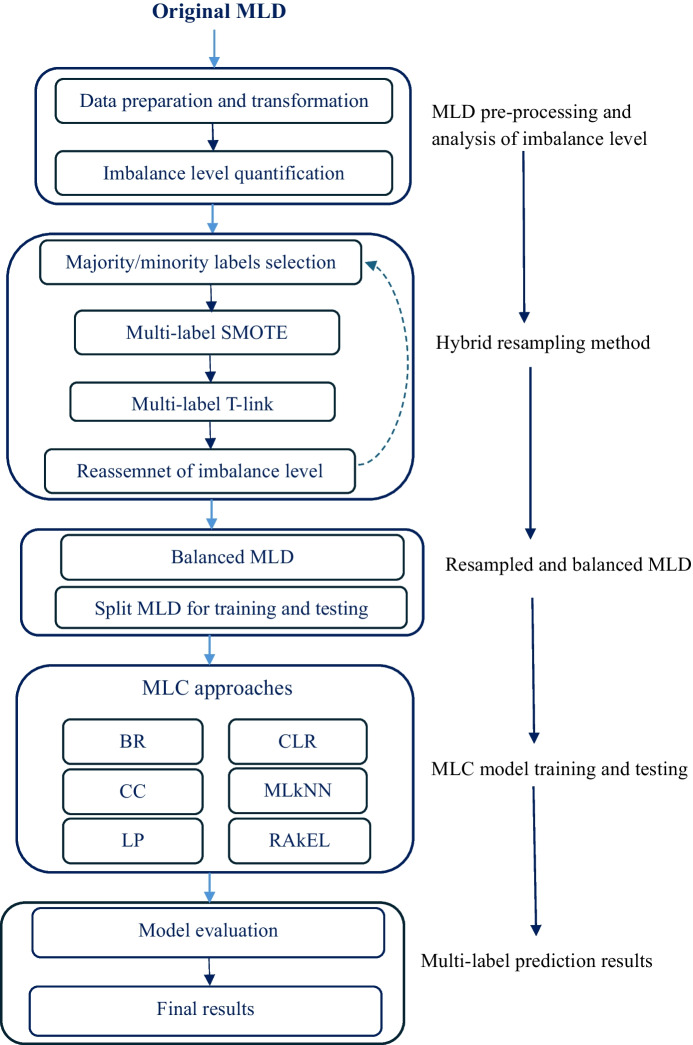
The proposed framework of multi-label-based frailty prediction

In this section, we present the proposed multi-label-based frailty prediction framework, briefly describing the data, analysis of label distributions and imbalance level in the data, the proposed hybrid resampling algorithm, multi-label classification methods along with evaluation measures and model development tools, as shown in Fig. 1.

### Data Source and Description

A detailed description of input and output variables in the data and related information is presented in [16]. Briefly, to develop a multi-label predictive model, we used health information retrieved from two years of administrative databases of older adults aged 65 years and above. Data were collected using an individual record linkage between the Italian 2011 census and the administrative health databases (enrollees' registry, hospital discharges, drug prescriptions, outpatient clinical investigation database, and health exemptions).

There are around 1 million anonymous record items consisting of input variables such as demographic, socioeconomic, and chronic conditions and output variables, which are described as outcomes or measurable changes in the health status of patients. In this study, six output variables that are associated with everyone's status are used as labels. They are mortality, urgent hospitalization, medical emergency admission at the emergency department, disability, fracture, and preventable hospitalization. This type of data is what we call a multi-label dataset (MLD). The way the data set is organized is such that one patient can have multiple outcomes.

### Label Distributions

All the six labels (i.e., the outcomes) in the data are binary-valued, as shown in Table 1, which presents some selected records from the original dataset. Labels that are associated with each record are called relevant (or active) labels, whereas the remaining (i.e., the non-associated labels) are the irrelevant ones. For example, in Table 1, labels 3, 5, and 6 are relevant to the first record, while labels 1, 2, and 4 are irrelevant ones (non-associated labels). Both the relevant and irrelevant labels are represented as a binary vector, with the size equal to the total number of labels in the data.

**Table 1:** An example of multi-label data records with the six labels

Records	Label 1	Label 2	Label 3	Label 4	Label 5	Label 6
${\text{r}}_{1}$	0	0	1	0	1	1
${\text{r}}_{2}$	0	0	1	1	1	0
${\text{r}}_{3}$	0	1	1	1	0	0
..	1	1	1	0	0	1
${\text{r}}_{\text{m}}$	1	1	1	1	1	0

1 points out the outcomes associated with each record, and *0* represents non-associated

We used label cardinality (Card) and label density (Dens) to describe the characteristics of our dataset. Label cardinality of a dataset M, denoted by card (M), is the average number of labels of examples in M. Label density of a dataset M, denoted by dens (M), is the average number of labels of examples in M divided by the number of labels. These measures are defined in Eqs. (10) and (11) [41], where =$\text{m}$|M| denotes the size of the dataset, ${|Y}_{i}|$ represents the number of labels for ${i}^{th}$ instance and |L| the number of labels in M. Table 2 shows the summary of the original dataset in terms of Card, Dens, number of input features (NF), the number of labels |L|, and the number of distinct label combinations (DC).

**Table 2:** Description of the multi-label dataset in the experiment

Dataset	Instances	NF	|L|	DC	Card	Dens
Frailty	1,095,613	58	6	64	0.133	0.022

10 $$\text{Card}\left(\text{M}\right)=\frac{1}{\text{m}}{\sum }_{\text{i}=1}^{\text{m}}\left|{\text{Y}}_{\text{i}}\right|$$

11 $$\text{Dens}\left(\text{M}\right)=\frac{1}{\text{m}}{\sum }_{\text{i}=1}^{\text{m}}\frac{\left|{\text{Y}}_{\text{i}}\right|}{\left|\text{L}\right|}$$

### Imbalance Levels

Based on the imbalance measures described in Sect. 2.2, the frailty dataset used in this paper has a MeanIR of 2.85 and a CVIR of 0.80, which shows that the dataset is imbalanced. Table 3 presents the imbalance ratio per label (IRLbl) of the six labels in the data. In this dataset, the most frequent (majority) labels are mortality and urgent hospitalization with an IRLbl of 1.0 approximately, whereas fracture and emergency admission are the less frequent (minority) ones with an IRLbl of 5.9 and 5.6, respectively.

**Table 3:** The imbalance level of each label in the frailty dataset

S.N	Labels	IRLbl
1	Mortality	1.000000
2	Urgent hospitalization	1.074644
3	Disability	1.330798
4	Preventable hospitalization	2.192901
5	Emergency admission	5.584591
6	Fracture	5.904701

### Proposed Resampling Approach

Several resampling approaches have been proposed to reduce the problem of imbalance in an MLC [24]. One of the main challenges of balancing label distribution through resampling methods is that adding new instances with minority labels also increases the frequency of labels, which are already majority ones. Similarly, removing instances from majority labels will lead to the loss of minority ones [27]. This problem has a strong impact on the resampling methods applied to our frailty dataset because, in most instances of the dataset, minority labels occur together with the majority ones. Thus, we proposed a new method by extending the existing approaches for solving the problem of imbalanced data in MLC suitable for the dataset we want to work with.

To solve this joint occurrence of majority and minority label distribution in the frailty dataset, we proposed a hybrid approach that combines SMOTE (synthetic minority oversampling) with Tomek links named ML-TLSMOTE (Multi-label SMOTE with Tomek links). ML-TLSMOTE can be used as a heuristic-based approach and combination of pre-processing methods whereby the SMOTE and Tomek links (T-link) cleaning methods are applied sequentially. SMOTE is applied first to generate synthetic instances of minority labels, and subsequently, T-link, which is used as a post-process cleaning step, is applied to the dataset composed of the original and new synthetic instances with majority labels. Each method and its hybrid version, which worked well for the traditional classification problem, is extended to the multi-label scenario to narrow down the gap between the most frequent labels and the least frequent ones as well as to reduce the imbalance between classes within each label.

To balance labels using ML-TLSMOTE, all instances that are both associated and non-associated with the current minority label are considered for SMOTE; at the same time, these instances should be non-associated with other label combinations. Then, T-link is applied for each majority label to make some adjustments between the classes of each label. The joint use of the SMOTE and T-link algorithms is designed to remove the imbalance between the labels and also reduce the imbalance within the labels.

#### Proposed Algorithm for ML-TLSMOTE

The detailed algorithm for the proposed hybrid approach (ML-TLSMOTE) is presented in **Algorithm 1.** The high-level description of the algorithm is summarized in four main parts in accordance with the following consecutive procedures:

a **Minority and Majority labels selection**: First, the set of minority labels and set of majority labels are identified from the MLD with the help of MeanIR and IRLbl. Labels with IRLbl less than MeanIR are considered majority labels and labels with IRLbl higher than MeanIR can be considered minority labels [27]. In Algorithm 1, lines from 1–4 handle the selection of minority labels, while lines from 6–12 select majority labels from the dataset based on the values of MeanIR and IRLbl.

**Algorithm 1:**
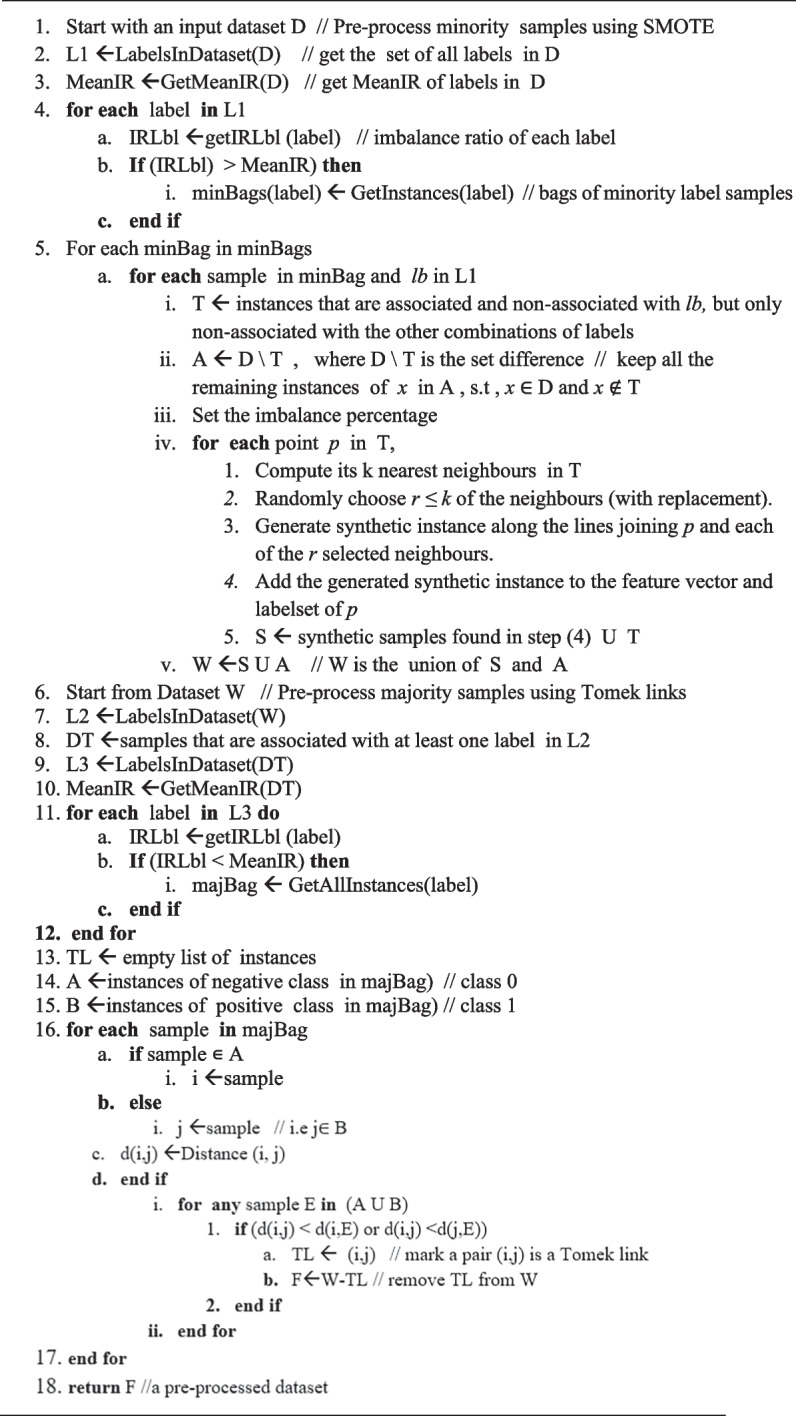
Proposed Algorithm for ML-TLSMOTE
b **Multi-label SMOTE**: Our MLD has more than one minority label. Therefore, each instance associated with each minority label (i.e., instances with 1’s for the minority label), but non-associated (value 0’s) with other label combinations are oversampled using SMOTE. Selecting active (associated) labels of minority instances and non-associated labels of majority instances help to increase only the frequency of rare labels without cloning the instances that are linked to the majority labels. In Algorithm 1, line 5a(i-v) computes multi-label SMOTE by taking inputs from the previous step.
c **Multi-label T-Link**: In this step, instances that are linked to the majority of labels are treated through the T-link cleaning method. T-link allows removing only the noisy or border samples of the majority labels. Removing T-link points can result in more well-defined class clusters in the training data, which can improve the performance of classifiers. As shown from lines 13–17 of the ML-TLSMOTE algorithm, we used T-link as a post-process cleaning step for two main reasons: (1) to reduce the imbalance between labels by removing instances that are associated with the majority labels, and (2) at the same time to clean up the non-associated instances of labels that were added as a result of the SMOTE preprocessing procedure (line 1-5v.), so that the imbalance within a label can be reduced or will not go to the extreme. In addition, after applying SMOTE on minority labels, the class groups of labels may not be well defined or overlapped due to the invasion of synthetic samples. Therefore, a data cleaning stage is desirable to clean up the borders between each class.
d **Reassess the imbalance level**: Finally, the IRLbl, MeanIR, and CVIR will be recalculated to check if the pre-processed MLD is balanced. At this stage, the MLD could have a more balanced label distribution and would be easier to process by the MLC algorithms. In Algorithm 1, line 18 returns a preprocessed and balanced dataset for further analysis using the MLC classifiers.

### MLC Methods and Performance Metrics

To predict frailty using the resampled data already, six different MLC algorithms were chosen [42]: Binary Relevance (BR), Classifier Chains (CC), Label Powerset (LP), Random k-label sets (RAkEL), Calibrated Label Ranking (CLR) and MLkNN. The classification results are evaluated using five multi-label metrics: Hamming loss, ranking loss, average precision, and label-based measures (F1score micro averaged, and AUROC macro averaged). The description of MLC methods and performance measures are presented in Sections. 2.1 and 2.3, respectively.

### Experimental Setup and Software Tools

The implementation and experiments were carried out on a personal computer with Intel(R) Core i7-1185G7 processor and installed memory (RAM) of 32 GB. The proposed resampling method was developed using Python version 3.7, while the experiments and evaluation of all MLC classifiers were carried out using the MEKA library [43]. MEKA is an open-source framework for multi-label learning and evaluation, which has been employed for the training and comparison of multi-label classifiers. We also used Rstudio statistical package for performing statistical test analysis for our experimental results.

The performance and efficiency of the proposed approach and MLC methods are highly dependent on the choice of a base classifier [44]. In order to select the best base classifiers for each MLC strategy (BR, CC, CLR, LP, MLkNN, and RakEL), we first consulted literature guidelines [44, 45] and performed preliminary experimental analysis. Based on this, we selected the commonly used base classifiers, namely random forest (RF), random tree (RT), decision tree (DT), support vector machine (SVM), and naïve Bayes (NB). The parameters of the base learners were set according to the recommendations in MEKA. For instance, when using SVM as the base learner, the following parameters were employed: the kernel was set to "polynomial" with a degree of 1 and the values chosen for C and gamma were 0.1 and 0.001, respectively. Next, we conducted further experimental studies on resampled datasets to determine the most effective base algorithm for each MLC strategy. These base classifiers are commonly used as hyperparameters for MLC strategies, with only one base algorithm used for training each MLC strategy [46]. For RAkEL, different values were set for the size of the label set (k = 2,3,4,5,6) and the number of models (m = 6,8,10,12,14,16) and the value k = 3 (default) and m = 12 were optimal. The parameters taken for the rest of the MLC methods were the default ones as suggested by their authors.

## Experimental Results

This section presents the experimental results of MLC methods achieved through the proposed ML-TLSMOTE approach. We split the results into two subsections, resampling results, and classification results.

### Resampling Results

For experimental analysis, we used 105,962 instances of an MLD, where each instance is associated with at least one active label of the label set. As already described in Section 3.2, the dataset contains six simultaneous adverse outcomes (i.e., labels) associated with frailty. Samples that are not associated with at least one active label in the dataset are excluded from the experiment. Moreover, the multi-label experimentation using MEKA has limited capability to handle the whole dataset. Once the resampling approaches were applied to the extracted MLD, the imbalance level of the pre-processed data was re-evaluated. Table 4 presents the imbalance level of the original MLD (i.e., the MLD without resampling, noted as Base), and the resampled MLD using T-link, SMOTE and ML-TLSMOTE, which is measured in terms of MaxIR, MeanIR, and CVIR values. The average imbalance level of the data after applying ML-TLSMOTE is MeanIR = 1.17 and CVIR = 0.12, which gives evidence that the imbalanced problem has been much reduced in the data as compared to SMOTE or T-link. The imbalance scores imply that the multi-label frailty dataset has a more balanced label distribution that can be further processed by the multi-label classification algorithms.

**Table 4:** Characteristics of the MLD before and after applying resampling algorithms

Resampling Methods	MaxIR	MeanIR	CVIR	Card	Dense
Without resampling (Base)	5.90	2.85	0.80	1.38	0.23
T-link (Under sampling)	1.70	1.42	0.18	0.50	0.08
SMOTE (Oversampling)	1.42	1.25	0.13	2.02	0.34
ML-TLSMOTE (Hybrid version)	**1.40**	**1.17**	**0.12**	**1.8**	**0.30**

From the results in Table 4, the behaviour of the original dataset has changed after applying the ML-TLSMOTE, which clearly shows that there is a general improvement in the imbalance levels. For the hybrid approach, the values of MeanIR and CVIR are below the threshold, which gives evidence that the ratio between the most frequent labels and the least frequent ones has been improved in the data. Thus, the MLD contain a more balanced label distribution that can be analyzed by the MLC methods.

### Classification Results via ML-TLSMOTE

Using the resampled MLDs in Table 4, several experiments were conducted using various MLC classification algorithms. To understand how the proposed hybrid resampling method (ML-TLSMOTE) has influenced the classification results, we used various multi-label classifiers, including BR, CC, LP, RAkEL, CLR, and MLKNN. The classification experiments were conducted using the resampled dataset as the training set and the non-resampled dataset as the test set. On the training dataset, a tenfold cross-validation was applied to train the MLC models. The proposed resampling algorithm was only performed on the training dataset, i.e., the dataset that was balanced, while the non-resampled test dataset which is representative of the original imbalanced dataset was used for the evaluation of classifiers.

Although the change in imbalance level will not necessarily imply better multi-label classification results, it has been observed that the lower the values of the imbalance levels, the better the performance of the MLC algorithms. However, there is an exception with the T-link method, where the performance of MLC classifiers on T-link was not improved. Table 5 presents the predictive performance of six different MLC classifiers using five multi-label metrics (AUROC, average precision, F1 score, Hamming loss and ranking loss) across the sampling approaches. The results show that CLR is the best model in terms of average precision and outperformed all other MLC classifiers via ML-TLSMOTE. It has also the best-ranking loss compared to the results obtained from the other classifiers. Table 5 also presents the standard deviation of each metric’s performance value across all models, which provides insights into the consistency or variability of the models' performance within each resampled dataset.

**Table 5:** Prediction performance for six MLC algorithms across the resampled datasets, with the best values highlighted in bold, and the standard deviation of each metric’s score across all models

Resampling Approaches	MLC Algorithms	MLC Measures*				
		AUROC	AP	F1-score	HL	RL
Hybrid proposed (ML-LSMOTE)	BR	**0.78**	**0.79**	0.68	0.17	0.19
	CC	0.76	0.62	0.68	0.18	0.28
	LP	0.76	**0.79**	**0.69**	0.17	0.20
	RAkEL	**0.83**	0.75	0.60	**0.15**	0.19
	CLR	0.81	**0.83**	0.67	0.20	**0.16**
	MLkNN	0.74	0.72	0.62	0.22	0.23
Standard deviation		0.033	0.059	0.029	0.019	0.029
Oversampling (SMOTE)	BR	0.73	0.73	0.54	0.20	0.21
	CC	0.71	0.64	0.55	0.17	0.29
	LP	0.73	0.70	0.58	0.17	0.23
	RAkEL	0.74	0.67	0.57	0.17	0.20
	CLR	0.78	0.75	0.59	0.22	0.16
	MLkNN	0.72	0.70	0.51	0.21	0.21
Standard deviation		0.021	0.029	0.018	0.012	0.05
Undersampling (T-link)	BR	0.55	0.58	0.40	0.27	0.32
	CC	0.55	0.65	0.41	0.35	0.37
	LP	0.57	0.50	0.44	0.30	0.36
	RAkEL	0.57	0.50	0.40	0.35	0.36
	CLR	0.56	0.53	0.37	0.43	0.41
	MLkNN	0.57	0.62	0.46	0.32	0.38
Standard deviation		0.012	0.034	0.018	0.065	0.023
Base (Without resampling)	BR	0.54	0.62	0.34	0.23	0.31
	CC	0.54	0.51	0.37	0.24	0.34
	LP	0.52	0.43	0.37	0.30	0.38
	RAkEL	0.57	0.57	0.46	0.33	0.31
	CLR	0.57	0.55	0.41	0.42	0.30
	MLkNN	0.57	0.64	0.34	0.27	0.26
Standard deviation		0.014	0.062	0.036	0.074	0.026

*AUROC (macro averaged), *AP*: average precision, F1-score (micro averaged), *HL*: Hamming loss, *RL*: Ranking loss

We also noticed that the use of different base classifiers for each MLC algorithm had shown a more significant effect on the variation of classification results on ML-TLSMOTE. In this study, five base classifiers (DT, RF, RT, SVM, NB) were evaluated on each MLC strategy, where BR, CC, and CLR have shown the best results when using the RT as a base classifier.

LP and RAkEL have achieved the best results when using SVM as the base classifier, while MLkNN used naïve Bayes as the best base classifier. However, we also found that using DT or RF as base classifiers resulted in relatively lower performance for all MLC methods. Overall, the results show that proposed method significantly outperforms traditional approaches and baseline across all metrics. For example, with F1 score, the BR algorithm via ML-TLSMOTE shows significant improvements over the SMOTE, T-link and baseline across all MLC algorithms, with performance increase from 5.26% to 25.93%.

## Discussions

This study framed the frailty problem into a multi-label learning task for the prediction of more than one adverse outcome simultaneously. This multi-label prediction problem is strongly motivated by the practical challenge of predicting several outcomes of frailty simultaneously from an imbalanced multi-label dataset. Although the single-label models (statistical or machine learning) for a clinical prediction problem have shown a strong predictive ability to estimate the risk of a single outcome associated with a disease condition [47–49], they are not well aligned to handle multiple outcomes simultaneously if the data originally contains multiple health outcomes. Moreover, the current studies on single-label classification for complex multi-label datasets fail to handle new approaches to improving performance through exploiting label correlations. The next section presents an analysis of experimental results aiming to detect more than one adverse outcome concurrently using the multi-label learning method.

### Analysis of Prediction Performance

The proposed ML-TLSMOTE method was evaluated considering the imbalanced multi-label dataset of older adults aged 65 years and above. Several experiments were conducted for testing the MLC algorithms using the proposed resampling method. Among the MLC algorithms, RAkEL, LP, and BR achieved the best performance in terms of the Hamming loss with ML-TLSMOTE (Table 5). RAkEL has shown the best performance in terms of macro average AUROC (83%) followed by the BR and LP with a score of 78% and 76%, respectively. CLR has achieved the best result in the average precision (83%). The Hamming loss captures the fraction of labels that are incorrectly predicted, while the ranking loss measures the average fraction of labels that are ordered incorrectly. For example, the ranking loss of RAkEL is 0.19, which means that 19% of the label pairs are wrongly ordered for instances. With the ranking evaluation measures, the CLR outperforms the other algorithms, which rank the relevant labels higher than irrelevant labels efficiently based on the pairwise comparison of labels. MLKNN showed poor performances in the Hamming loss and the ranking loss as compared to the BR, RAkEL, and LP.

For the concrete establishment of the best model across all resampled datasets, we calculated the ranking of each model according to their average precision, where the average rank of each model was calculated using the formula:

$${R}_{i} =\frac{1}{N}\sum_{i=1}^{N}{S}_{i}^{j}$$

where ${S}_{i}^{j}$ is the rank of i^th^ model for the j^th^ resampled dataset. The calculated average ranks of the models in all resampled datasets are shown in Table 6. The results can be observed that BR and CLR have the lowest average rankings score across all datasets, which means that they are the best-performing classifiers when measured with average precision, while CC has a higher average ranking which indicates it is consistently performing poorly in all resampled datasets. CLR is also the best-performing model on ML-TLSMOTE and SMOTE, while CC is the worst classifier.

**Table 6:** Average ranks of MLC models for frailty prediction based on their average precision over SMOTE, T-link, ML-TLSMOTE, and Base datasets

	Average Rank of MLC Models					
Datasets	BR	CC	LP	RAkEL	CLR	MLkNN
Base	2	5	6	3	4	1
T-link	3	1	5.5	5.5	4	2
SMOTE	2	6	3.5	5	1	3.5
ML-TLSMOTE	2.5	6	2.5	4	1	5
Average Rank	**2.38**	**4.5**	**4.38**	**4.38**	**2.5**	**2.88**

In addition, to highlight the efficacy of our proposed algorithm, a non-parametric Friedman aligned ranking (FAR) [50] and Wilcoxon signed rank test are carried out, following literature guidelines [51]. Both tests are performed in terms of F1-score (micro averaged) where FAR is applied across all the six MLC methods using multiple comparison procedures [52], and Wilcoxon signed rank test is performed to check the significance of the difference between ML-TLSMOTE and other resampling algorithms. Table 7 presents the ranking of the proposed resampling algorithm using FAR and the pairwise comparison results according to the Wilcoxon signed rank test with α = 0.05.

**Table 7:** Friedman aligned ranking (FAR) and Wilcoxon signed-rank test

Algorithms	FAR	Algorithms	Wilcoxon signed-rank test	
			*P*-value	Null hypothesis
ML-TLSMOTE	1.13	-	-	-
SMOTE	2.17	ML-TLSMOTE vs. SMOTE	0.036	Rejected
T-link	3.33	ML-TLSMOTE vs T-link	0.031	Rejected
Base	3.67	ML-TLSMOTE vs. Base	0.030	Rejected

As shown in Table 7, statistical analysis using F1-score (micro averaged), and other metrics (AUROC and average precision) have been performed to measure the significance of differences between the ML-TLSMOTE and other single-label approaches. The statistical results show evidence that ML-TLSMOTE achieved the highest statistical ranking with higher classification performance of MLC methods in the frailty problem. The performance results of the MLC approach via ML-TLSMOTE are in line with the statistical test result (FAR = 1.13) ranking. In addition, the pairwise test using Wilcoxon signed rank test shows the significance of the difference between ML-TLSMOTE and other methods (SMOTE, T-link and the Base).

Overall, from the analysis of results, it can be concluded that the proposed strategy (I.e., ML-TLSMOTE) is an effective approach for solving an imbalanced MLC and has a more positive influence over all the multi-label classifiers, which enhances the prediction of multiple outcomes associated with frailty syndrome.

### Computational Complexity

Finally, with efficiency measures, the computational complexity of the BR, CLR, and LP depends on the complexity of the base classifier and the parameters of the learning problem [44]. We observed that using tree-based methods as a base classifier (e.g., C4.5) is more efficient than using the SVM-based methods. The BR algorithm, which builds separate models for each label associated with frailty, is the simplest one. In our experiment for the data preprocessed with ML-TLSMOTE, the training time of BR using C4.5 as the base classifier was 20.24 min, while the training time of BR using SVM was 3.5 h. The CLR is the next least complex algorithm, requiring |L| the number of BR models and additionally |L|*(|N|-1)/2 one against one model. Through ML-TLSMOTE, the training time of CLR using a random tree as the base classifier was 8.5 min. The LP is relatively the most sophisticated algorithm, since it trains a multi-class classifier, with the number of classes being equal to the number of distinct label sets in the MLD. The computational complexity of MLKNN is |L| times the computational cost of computing K nearest neighbours. The training MLKNN model is linear with the size of the training dataset and the length of the data vector.

The main advantage of our proposed resampling approach (ML-TLSMOTE) is that it is independent of both the multi-label classifiers and the base algorithms. Thus, it does not demand training any of these classification algorithms and can be used as a general solution to the problem. Regardless of the imbalanced solution proposed in this study, any multi-label learning problem has additional complexities due to the presence of a large number of labels, high multidimensionality and concurrency of imbalanced labels. ML-TLSMOTE is developed to solve the concurrency of imbalanced labels and to reduce imbalance within labels. Generally, concurrency is a more complicated problem as the number of labels increases, where the proposed ML-TLSMOTE approach can help to handle the challenge of concurrency between labels.

## Conclusions

Detecting frailty in elderly people represents an essential research problem, and there is a potential to prevent frailty and intervene early. In this study, MLC was developed for the purpose of predicting multiple outcomes of frailty conditions: mortality, fracture, disability, medical emergency admission at the emergency department, urgent hospitalization, and preventable hospitalization. MLC models are valuable tools to construct a predictive model that considers the prediction of multiple outcomes and interventions in an unseen patient. The study consists of two major points: the first is addressing the imbalance problem in an MLC. ML-TLSMOTE was proposed to reduce the imbalance between labels and improve the performance of MLC algorithms for frailty prediction. The results of the experiment show that ML-TLSMOTE was an efficient approach as compared to SMOTE or T-link. The second part presents a comparative study of the six MLC algorithms (BR, ECC, LP, CLR, RAkEL, and MLKNN) for the prediction of frailty. RAkEL achieved the best performance in terms of the Hamming loss and macro averaged AUROC, while the CLR showed the best value of the ranking loss and average precision.

In future work, three problems need further investigation in our study. The first is dimensionality reduction to optimize and improve the performance of the training models, which is one of the challenging topics in the MLC task. Second, with the advances in sensor technologies, many elderly people with frailty can use wearable sensors [53–55] to monitor their physiological signals; thus, it is essential to collect and analyze real-time data from wearable sensors to make a more accurate frailty risk assessment. Finally, we need to apply and test ML-TLSMOTE on other benchmark multi-label databases, such as images, and textual datasets using advanced deep learning models [56] and evaluate its performance and computational complexity.

## Data Availability

No datasets were generated or analysed during the current study.
